# Dermatologic and Dermatopathologic Features of Monogenic Autoinflammatory Diseases

**DOI:** 10.3389/fimmu.2019.02448

**Published:** 2019-10-29

**Authors:** Ignasi Figueras-Nart, José M. Mascaró, Xavier Solanich, José Hernández-Rodríguez

**Affiliations:** ^1^Department of Dermatology, Bellvitge Hospital, University of Barcelona, Barcelona, Spain; ^2^Department of Dermatology, Hospital Clinic, IDIBAPS, University of Barcelona, Barcelona, Spain; ^3^Department of Internal Medicine, Bellvitge Hospital, University of Barcelona, Barcelona, Spain; ^4^Clinical Unit of Autoinflammatory Diseases and Vasculitis Research Unit, Department of Autoimmune Diseases, Hospital Clinic, IDIBAPS, University of Barcelona, Barcelona, Spain

**Keywords:** monogenic autoinflammatory diseases, autoinflammatory diseases, clinical dermatology, maculopapular rash, urticarial rash, dermatopathology, classification

## Abstract

Autoinflammatory diseases include disorders with a monogenic cause and also complex conditions associated to polygenic or multifactorial factors. An increased number of both monogenic and polygenic autoinflammatory conditions have been identified during the last years. Although skin manifestations are often predominant in monogenic autoinflammatory diseases, clinical and histopathological information regarding their dermatological involvement is still scarce. Monogenic autoinflammatory diseases with cutaneous expression can be classified based on the predominant lesion: (1) maculopapular rashes or inflammatory plaques; (2) urticarial rashes; (3) pustular, pyogenic or neutrophilic dermatosis-like rashes; (4) panniculitis or subcutaneous nodules; (5) vasculitis or vasculopathy; (6) hyperkeratotic lesions; (7) hyperpigmented lesions; (8) bullous lesions; and (9) aphthous lesions. By using this classification, this review intends to provide clinical and histopathological knowledge about cutaneous involvement in monogenic autoinflammatory diseases.

## Introduction

The term “autoinflammatory diseases” was first used in 1999 to describe a group of rare diseases of the innate immunity presenting with recurrent episodes of uncontrolled systemic inflammation (1). Since then, the number of monogenic autoinflammatory conditions and other complex and polygenic disorders driven by autoinflammatory mechanisms have been in continuous expansion (2, 3). In addition, several autoimmune diseases and primary immunodeficiencies have been found to share pathogenic features with autoinflammatory diseases (4, 5).

The most frequent and well-known autoinflammatory mechanism is mediated by the inflammasomes, intracellular protein complexes acting as innate immune system receptors with an important role in the sensing of intracellular pathogen- and danger-associated molecular patterns. They are involved in the susceptibility to infection, autoinflammation, and tumorigenesis. Inflammasomes consist of a sensor part (the NOD-like-receptor), an adaptor protein (ASC), and caspase-1 as the downstream effector. Upon stimulation, inflammasome assembles and activates caspase-1 which cleaves pro-IL-1β and pro-IL-18 into IL-1β and IL-18. NRLP3 and pyrin inflammasomes are responsible for cryopyrin-associated periodic syndromes (CAPS) and familial Mediterranean fever (FMF), respectively, and other inflammasomopathies (6, 7). Other relevant inflammasomes include NLRP1 and NLRP4 (8, 9).

Other pathogenic mechanisms causing autoinflammatory disorders include those related with the activation of NF-κB transcription factor and type I interferon (IFN) (6, 10). The transcription factor NF-κB is involved in processes related to inflammation, cellular differentiation, metabolism, cell survival, and acquired immune responses (6). In its inactive form, NF-κB is tied to inhibitors of kBs (IkBs). NF-κB can be activated by two mechanisms: the canonical pathway, induced by cytokines and toll-like receptors (TLR), and the non-canonical pathway, triggered by TNF-receptor family proteins. Both are controlled by the ubiquitin system. The canonical mechanism is regulated by K63 and linear Met1 ubiquitin chains. Both proteins are linked to their substrates (RIPK-1, which is one of the adaptor proteins on the TNF receptor 1, and IKKγ, part of the IKK complex) by LUBAC complex (composed by the proteins HOIP, HOIL-1, and SHARPIN), which increases NF-κB activity. Proteins A20 and OTULIN cleave K63 and Met1 from their substrates, which physiologically downregulate NF-κB signaling. Little is known about its exact role in the non-canonical pathway (10–12).

Type I interferons (IFNα and IFNβ) are the major effector cytokines against virus and intracellular pathogens. They induce the transcription of certain IFN stimulated genes with the subsequent viral clearance. Among the two IFN activating mechanisms, one is mediated by TLRs that detect viral nucleic acids within endosomes and induce proinflammatory cytokines and IFNα, and the other is mediated by cytosolic DNA and RNA sensors. DNA sensing is carried out by nucleotidyl transferase cyclic GMP-AMP synthase (cGAS), which produces cGAMP that binds to STING (stimulator of IFN genes) and induces transcription of IFNβ genes. RNA sensing is mediated by RIG-1-like helicase, RIG-1, and MDA-5 with the subsequently recruitment of MAVS (mitochondrial antiviral signal) and activation of NF-κB. IFN interacts with its surface receptor IFN-α and induces the STAT pathway, which induces the transcription of IFN genes and promotes antiviral activity. In addition, proteins regulating the synthesis or degradation of nucleic acids such as TREX1, SAMHDI, and RNase H2 play an important role in IFN genes activation. Immunoproteasomes are protein complexes that degrade ubiquitinated intracellular proteins and are implicated in cellular stress responses, as well as activating IFN (11, 13).

**Figure 1:**
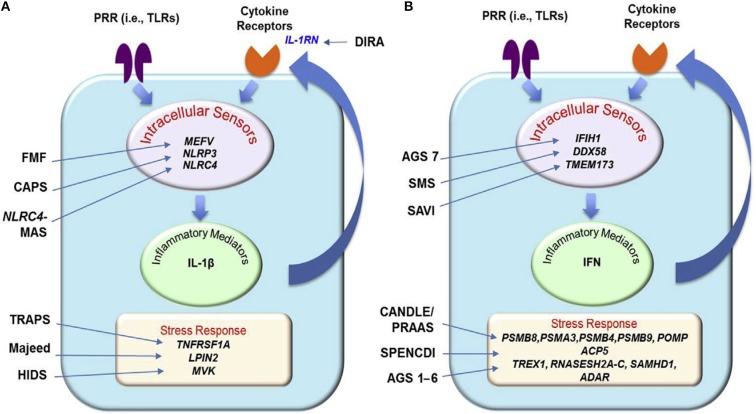
Principal genetic and pathogenic mechanisms in IL-1 **(A)** and IFN type 1 **(B)** mediated autoinflammatory diseases [From Shwin et al. (14), with permission]. AGS, Aicardi–Goutières syndrome; CANDLE, chronic atypical neutrophilic dermatosis with lipodystrophy and elevated temperature; CAPS, cryopyrin-associated periodic syndrome (FCAS, familial cold autoinflammatory syndrome; MWS, Muckle–Wells syndrome; NOMID, neonatal-onset multisystem inflammatory disease); DIRA, deficiency of interleukin-1 receptor antagonist; FMF, familial Mediterranean fever; MKD/HIDS, mevalonate kinase deficiency/hyperimmunoglobulinemia D and periodic fever syndrome; NLRC4-MAS, NLRC4-associated macrophage activation syndrome; PRAAS, proteasome-associated autoinflammatory syndrome; PRR, Pattern recognition receptor; SAVI, STING-associated vasculopathy with onset in infancy; SMS, Singleton–Merten syndrome; SPENCDI, Spodyloenchondrodysplasia with immune dysregulation; TLRs, toll-like receptors; TRAPS, TNF receptor-associated periodic syndrome.

IL-1-mediated and IFN type I-mediated autoinflammatory diseases and their main genetic and pathogenic aspects are illustrated in Figure 1.

Over time, different classifications of monogenic autoinflammatory diseases have been proposed according to molecular and etiopathogenic mechanisms involved (11, 15), type of inheritance (16), genetic background and clinical presentation (17, 18). Apart from FMF and CAPS, other well-characterized monogenic inflammasomopathies comprise TNF receptor-associated periodic syndrome (TRAPS), hyper-IgD syndrome (HIDS), pediatric granulomatous arthritis (Blau syndrome and early onset sarcoidosis), pyogenic arthritis, pyoderma gangrenosum, and acne (PAPA), deficiency of IL-1 receptor antagonist (DIRA) and deficiency of interkeukin-36 receptor antagonist (DITRA). All the monogenic autoinflammatory diseases covered in this review classified according to the major pathogenic mechanism are listed in Table 1.

**Table 1:** Classification of autoinflammatory diseases based on the major pathogenic mechanism.

**Groups based on pathogenic mechanism**	**Autoinflammatory disease**	**Gene/Locus**	**Inheritance pattern**	**Protein involved**	**GOF/LOF mutation**
Inflammasomopathies	FMF	*MEFV*	AR	Pyrin	GOF
	TRAPS	*TNFRSF1A*	AD	TNF receptor 1	LOF
	HIDS/MKD	*MVK*	AR	Mevalonate kinase	LOF
	CAPS	*NLRP3*	AD	NLRP3/cryopirin	GOF
	NLRC4-AD (FCAS4)	*NLRC4*	AD	NLRC4	GOF
	PAPA	*PSTPIP1*	AD	CD2BP1	GOF
	DIRA	*IL1RN*	AR	IL-1 receptor antagonist	LOF
	Majeed syndrome	*LPIN2*	AR	Lipin-2	LOF
	PAAND	*MEFV*	AD	Pyrin	GOF
	NAIAD	*NLRC1*	AR/AD	NLRP1	LOF
	PFIT	*WDR1*	AR	WD40 repeat protein	LOF
	CAIN	*CEBPE*	AR	C/EBPε	GOF
NF-κB related diseases	Blau syndrome/Early-onset sarcoidosis	*NOD2/CARD15*	AD	NOD2	GOF
	NLRP12-AD (FCAS2)	*NLRP12*	AD	Monarch1	LOF
	Otulipenia/ORAS (Ubiquinopathy)	*OTULIN*	AR	Otulin	LOF
	HA20 (Ubiquinopathy)	*TNFAIP3*	AD	A20	LOF
	HOIL-1 deficiency (Ubiquinopathy)	*HOIL1*	AR	HOIL1	LOF
	CARD-14 psoriasis	*CARD14*	AD	CARD14	GOF
	NFKB1-AD	*NFKB1*	AD	p50/p105	LOF
	RELA haploinsufficiency	*RELA*	AD	RelA	LOF
	ADAM17 deficiency	*ADAM17*	AR	TACE	LOF
Interferonopathies	CANDLE/PRAAS syndrome	*PSMB8*	AR	β5i subunit of the proteasome	LOF
	SAVI	*TMEM173*	AD	STING	GOF
	Familial chilblain lupus	*TREX1 SAMHD1 TMEM173*	AD	3-prime repair exonuclease 1 enzyme dNTPs STING	LOF LOF GOF
	AGS	*TREX1, RNASEH2A, RNASEH2B, RNASEH2C and SAMHD1*	AR	Proteins involved in intracellular degradation or sensing of nucleic acids	LOF > GOF
		*ADAR1 IFIH1 and DDX58*	AD		
	SPENCDI	*ACP5*	AR	TRAP	LOF
	SMS	*IFIH1 and DDX58*	AD	MDA5 and RIG-1	GOF
Other	DADA2	*CECR1*	AR	ADA2	LOF
cytokine-signaling	DITRA	*IL36RN*	AR	IL-36 receptor antagonist	LOF
diseases	H syndrome	*SLC29A3*	AR	hENT3	LOF
	PLAID (FCAS3) / APLAID	*PLCγ2*	AD	PLCγ2	GOF
	Vibratory Urticaria	*ADGRE2*	AD	ADGRE2	LOF
	AP1S3 and autoinflammatory psoriasis	*AP1S3*	Not clear	AP1S3	LOF
	Monogenic forms of inflammatory bowel disease (IL-10 signaling defects)	*IL10RA, IL10RB and IL10*	AR	IL10 and IL10 receptor	LOF

*AGS, Aicardi-Goutières syndrome; APLAID, autoinflammation and PLCγ2-associated antibody deficiency and immune dysregulation; CAIN, C/EBPε-associated autoinflammation and immune impairment of neutrophils; CANDLE, chronic atypical neutrophilic dermatitis with lipodystrophy and elevated temperature syndrome; CAPS, cryopyrin-associated periodic syndrome; DADA2 deficiency of adenosine deaminase 2; DIRA, deficiency of IL-1 receptor antagonist; DITRA, deficiency of the IL-36 receptor antagonist; dNTPs, deoxynucleoside triphosphate; FMF, familial Mediterranean fever; GOF, gain-of-function; HA20, haploinsufficiency of A20; HIDS/MKD, hyper-IgD syndrome/Mevalonate kinase deficiency; IL-10, interleukin 10; LOF, loss-of-function; MDA-5, melanoma differentiation-associated gene 5; NFKB1-AD, NFKB1-associated autoinflammatory diseases; NLRC4-AD, NLRC4-associated autoinflammatory diseases; NLRP12-AD = NLRP12-associated autoinflammatory disease; ORAS = OTULIN-related autoinflammatory syndrome; PAAND, pyrin-associated autoinflammation with neutrophilic dermatosis; PAPA syndrome, pyogenic sterile arthritis, pyoderma gangrenosum and acne syndrome; PFAPA, periodic fever, aphthous stomatitis, pharyngitis and cervical adenitis; PFIT, Autoinflammatory periodic fever, immunodeficiency, and thrombocytopenia; PLAID, PLCγ2-associated antibody deficiency and immune dysregulation; RIG-1, retinoic-acid-inducible gene; SAVI, STING-associated vasculopathy with onset in infancy; SMS = Singleton-Merten syndrome; SPENCDI, Spondyloenchondrodysplasia with immune dysregulation; TRAPS, TNF receptor-associated periodic syndrome*.

Polygenic or multifactorial autoinflammatory diseases are defined as complex systemic disorders sharing an autoinflammatory and sometimes autoimmune background, with an unknown genetic cause. The most prevalent polygenic conditions include Behçet disease, Schnitzler syndrome, periodic fever with aphthous stomatitis, pharyngitis, and cervical adenitis (PFAPA), systemic juvenile idiopathic arthritis (sJIA), adult onset Still disease (AOSD), Crohn disease and synovitis, acne, pustulosis, hyperostosis, and osteitis (SAPHO) (19).

Clinical features in autoinflammatory diseases are variable, heterogeneous and nonspecific, since most of the symptoms are often shared by different conditions. Common inflammatory manifestations include recurrent fever, musculoskeletal symptoms, abdominal and thoracic serositis, headache, ocular inflammation, and mucosal and skin lesions (11).

Dermatologic involvement is common in monogenic autoinflammatory diseases and may represent the predominant and the initial event in some of them. Among all the cutaneous lesions present in monogenic autoinflammatory diseases, maculopapular, and urticarial rashes are by far the most prevalent manifestations. However, the identification of skin lesions as part of an autoinflammatory disease is often difficult because of the potential wide spectrum of skin manifestations in these conditions, and also because the severity or extension of the cutaneous lesions may differ among patients with the same disease. In addition, some patients may exhibit overlapping skin manifestations. Consequently, differential diagnosis of dermatologic findings may be difficult, even for trained professionals. For instance, with regard to urticarial lesions, differential diagnosis should include all CAPS forms and other monogenic diseases in which urticariform features are the most characteristic cutaneous findings, but it must also comprise other monogenic autoinflammatory diseases presenting less frequently with urticarial rashes (e.g., TRAPS and HIDS), and several polygenic autoinflammatory diseases (e.g., Schnitzler syndrome, sJIA, and adult onset Still disease) (20). Moreover, clinical and histopathological data about dermatological involvement in monogenic autoinflammatory diseases are still scarce (14).

## Classification of Monogenic Autoinflammatory Diseases According to the Cutaneous Involvement

Several classifications based on clinical and histopathological features of cutaneous manifestations have been proposed for autoinflammatory diseases (6, 11, 15, 16, 21, 22). In 2017, Shwin et al. (14) divided monogenic autoinflammatory diseases into seven categories according to the predominant cutaneous lesion and the most clinically relevant aspect: (1) Nonspecific maculopapular rashes with recurrent episodic fever and abdominal pain; (2) Neutrophilic urticaria; (3) Pustular skin rashes and episodic fevers; (4) Vasculopathy and panniculitis/lipoatrophy syndromes; (5) Vasculopathy and/or vasculitis with livedo reticularis syndromes; (6) Autoinflammatory disorders with granulomatous skin diseases; and (7) Other autoinflammatory syndromes (14).

Because other cutaneous and mucosal lesions have been described to occur in monogenic autoinflammatory diseases, the current review propose a new classification that includes nine dermatologic categories:

1. Maculopapular rashes or inflammatory plaques;
2. Urticarial rashes;
3. Pustular, pyogenic, or neutrophilic dermatosis-like rashes;
4. Panniculitis or subcutaneous nodules;
5. Vasculitis or vasculopathy;
6. Hyperkeratotic lesions;
7. Hyperpigmented lesions;
8. Bullous lesions;
9. Aphthous lesions.

The main monogenic autoinflammatory diseases are divided in these nine groups and depicted in Table 2. By using this dermatologic classification, this review intends to focus on dermatological and dermatopathologic aspects of monogenic autoinflammatory diseases.

**Table 2:** Classification of monogenic autoinflammatory diseases based on the main cutaneous manifestation.

1	Maculopapular rashes or inflammatory plaques	Familial Mediterranean Fever (FMF)
		TNF receptor-associated periodic syndrome (TRAPS) Hyper-IgD syndrome/Mevalonate kinase deficiency (HIDS/MKD) Otulipenia/OTULIN-related autoinflammatory syndrome (ORAS) HOIL-1 deficiency
2	Urticarial rashes	Cryopyrin-associated periodic syndromes (CAPS) NLRP12-associated autoinflammatory disease (NLRP12-AD) PLCγ2-associated antibody deficiency and immune dysregulation (PLAID) NLRC4-associated autoinflammatory diseases (NRLC4-AD) Vibratory Urticaria
3	Pustular, pyogenic or neutrophilic dermatosis-like rashes	Pyogenic sterile arthritis, pyoderma gangrenosum and acne (PAPA) Syndromic forms of pyoderma gangrenosum Deficiency of IL-1 receptor antagonist (DIRA) Deficiency of IL-36 receptor antagonist (DITRA) CARD-14 mediated psoriasis (CAMPS) Majeed syndrome Pyrin-associated autoinflammation with neutrophilic dermatosis (PAAND) Singleton-Merten syndrome (SMS) ADAM17 deficiency AP1S3 and autoinflammatory psoriasis NFKB1-associated sterile familial autoinflammatory necrotizing fasciitis (FANF)
4	Panniculitis or subcutaneous nodules	Blau syndrome / Early-onset sarcoidosis Chronic atypical neutrophilic dermatitis with lipodystrophy and elevated temperature (CANDLE)
5	Vasculitis or vasculopathy	Deficiency of adenosine deaminase 2 (DADA2) STING-associated vasculopathy with onset in infancy (SAVI) Familial chilblain lupus Aicardi-Goutières syndrome (AGS) 1-7 Spodyloenchondrodysplasia with immune dysregulation (SPENCDI)
6	Hyperkeratotic lesions	NLRP-1 associated disease (NAIAD)
7	Hyperpigmented lesions	H syndrome
8	Bullous lesions	Autoinflammation and PLCγ2-associated antibody deficiency and immune dysregulation (APLAID)
9	Aphthous lesions	Haploinsufficiency of A20 (HA20) Autoinflammatory periodic fever, immunodeficiency and thrombocytopenia (PFIT) C/EBPε-associated autoinflammation and immune impairment of neutrophils (CAIN) NFKB1-associated Behcet-like disease RELA haploinsufficiency Monogenic forms of inflammatory bowel disease (IL-10 signaling defects)

### Maculopapular Rashes or Inflammatory Plaques

#### Familial Mediterranean Fever (FMF)

FMF is the most frequent monogenic autoinflammatory disease caused by mutations in the *MEFV* gene, which encodes pyrin. Such mutations produce a constitutive activation of pyrin and lead to an uncontrolled release of IL-1β and IL-18 (23). FMF is classically inherited with an autosomal recessive fashion. However, an autosomal dominant pattern has also been described (24, 25). The most relevant pathogenic mutations, such as M694V, M694I, M680I, and V726A, are commonly placed in the exon 10 of *MEFV* gene (26).

FMF is clinically characterized by recurrent and self-limited inflammatory attacks lasting for 48–72 h with a variable periodicity (27). High fever (38–40°C) and serositis as abdominal and chest pain are constantly present. Large joints involvement and erysipeloid rash affecting the limbs are also quite common. Febrile protracted myalgia, pericarditis, scrotal pain, and lymphocytic meningitis may also occur. During attacks, acute phase reactants such as C-reactive protein (CPR), serum amyloid protein (SAA), erythrocyte sedimentation rate (ESR), and fibrinogen are significantly increased and tend to normalize during asymptomatic periods. Secondary amyloidosis, usually involving the kidneys, is the most common long-term complication, which is usually associated with a more severe disease or colchicine-resistant disease (14, 26).

Colchicine is the treatment of choice to control disease activity and to prevent the attacks. Colchicine also prevents the development of amyloidosis. In cases of proved intolerance or resistance to colchicine, anti-IL-1 agents have demonstrated efficacy in controlling disease activity and amyloidosis development. While canakinumab has been recently approved by the US Food and Drug Administration (FDA) and European Medicines Agency (EMA) (28), anakinra has also been proved to be useful, either with a continuous or on demand administration (29).

##### Dermatologic manifestations

**Figure 2:**
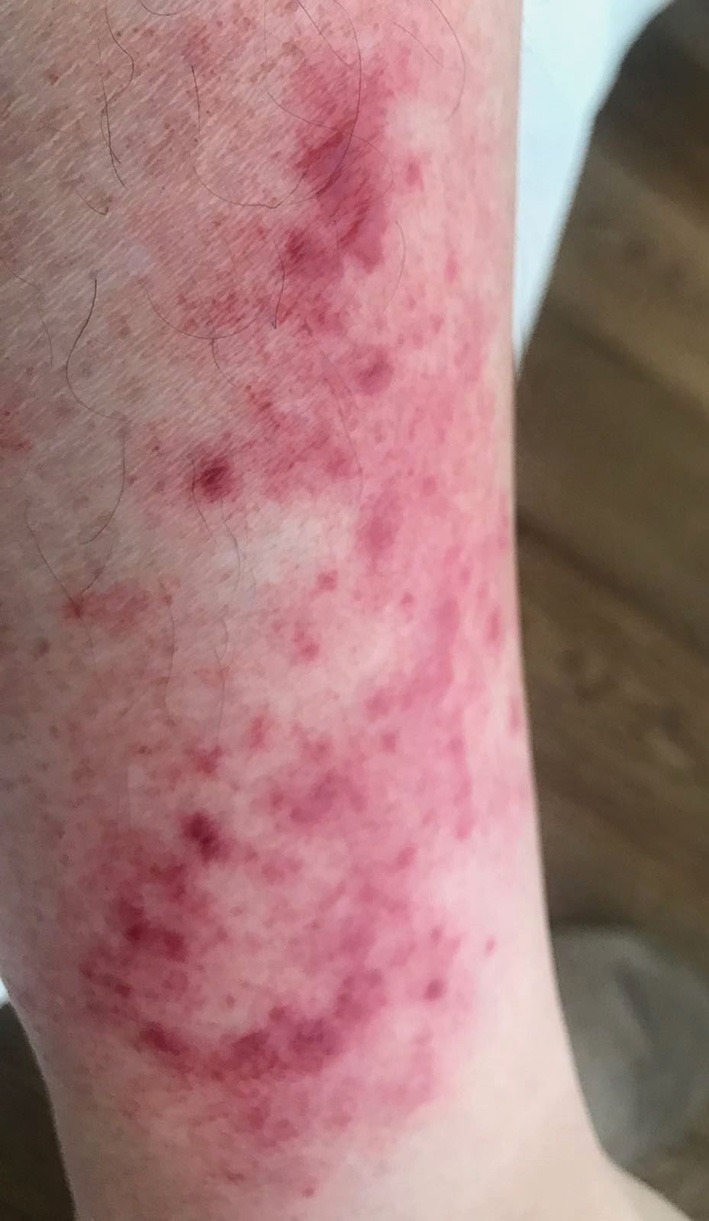
Erysipeloid lesion in a leg of a patient with FMF. Written informed consent was obtained from the patient for the publication of this image.

Erysipeloid-like erythema is considered the pathognomonic lesion of FMF and consists of an uni- or bilateral well-defined, tender, erythematous, and edematous plaque, usually smaller than 15 centimeters, localized below the knee and on the dorsal aspect of the feet (Figure 2). Recurrences tend to occur in the same place, usually after long walking distances, and tend to subside within 24–48 h. It is common among Turks and Jews patients and those carrying the M694V mutation, with a variable frequency, ranging between 3 and 46% of FMF patients (30).

Other cutaneous lesions include diffuse palmoplantar erythema and purpuric papules involving the face, trunk, and extremities (31). FMF patients have an increased incidence of associated systemic vasculitis, such as IgA vasculitis (Henoch-Schönlein purpura), polyarteritis nodosa and Behçet disease (31, 32).

##### Cutaneous histopathology

Erysipeloid-like plaques are histologically characterized by slight edema of the superficial dermis and sparse perivascular infiltrates with lymphocytes, neutrophils, histiocytes, and nuclear dust. Blurring of the capillary walls is frequent. Direct immunofluorescence shows deposits of IgM, C3, and fibrinogen in the capillary walls of the papillary dermis (30). Slight changes of acanthosis and hyperkeratosis in the epidermis have also been described (33).

#### TNF Receptor-Associated Periodic Syndrome (TRAPS)

TRAPS is the most frequent autosomal dominant autoinflammatory disease. Mutations in the *TNFRSF1A* gene, encoding TNF receptor 1, induce an overproduction of IL-1β (11). T50M and cysteine mutations are associated with an earlier and more severe disease presentation and long-term development of complications, such as amyloidosis. Variants such as R92Q and P46L generally lead to a milder disease with a later onset (2).

TRAPS usually occurs in children as recurrent and irregular febrile episodes with generalized myalgia, arthralgia, abdominal pain, ocular lesions (conjunctivitis, uveitis, and periorbital edema) and skin involvement (16, 34). Attacks may be spontaneous or triggered by infections and other stress situations (35).

Acute phase reactants, including CRP, ESR, and ferritin, are usually increased during attacks and subside after them. Secondary amyloidosis may occur in 25% of patients, mostly in those untreated (14, 34).

On demand use of non-steroidal anti-inflammatory drugs (NSAID) and glucocorticoids during attacks may improve symptoms in 40% of patients. With regard to anti-TNF agents, etanercept is the only proving efficacy in controlling attacks, since infliximab, and adalimumab have been associated with severe paradoxical reactions. IL-6 blockade with tocilizumab may also be of benefit in some cases. IL-1 inhibition seems to be the treatment of choice in TRAPS patients (36, 37). Anakinra is effective in most cases, administered either continuously or on demand (38), and canakinumab has been recently approved by the FDA and the EMA as first line therapy (28).

##### Dermatologic manifestations

About 80% of patients present with skin lesions. The most frequent is the painful erythema that consists of a migratory, centrifugal, erythematous, tender, non-purpuric, and well-demarcated plaque overlying migratory myalgia. The differential diagnostic of these erythematous lesions comprises cellulitis plaque or panniculitis of the limbs (14–16). Other manifestations include (16) urticaria-like plaques, generalized serpiginous plaques, and small-sized vessel vasculitis (16, 32, 39, 40).

##### Cutaneous histopathology

Histological specimens of TRAPS are characterized by a mild to massive perivascular and interstitial lymphocytic and monocytic infiltrate (CD3+, CD4+, CD8+, CD68+, CD79a–, and CD20–) in edematous areas of the superficial and deep dermis, with no evidence of multinucleated macrophages nor granulomatous or leukocytoclastic vasculitis. Direct immunofluorescence reveals deposits of IgM and C3 at the dermal-epidermal junction or diffuse interstitial deposits of IgA, G, and C3. Perivascular C3 and C4 deposition in the dermis is also described (14, 16, 34, 39, 41).

#### Hyper-IgD Syndrome (HIDS)

HIDS and mevalonic aciduria (MA) represent parts of the spectrum of the mevalonate kinase (MVK) deficiency (MKD) (2, 42). Both diseases are inherited with an autosomal recessive pattern and caused by mutations in the *MVK* gene, which encodes MVK, an enzyme involved in the synthesis of non-steroidal isoprenoids and also in the caspase activation pathway (14, 43–45). The amount of residual enzymatic activity correlates inversely with phenotype severity. V377I and I268T are the most frequent pathogenic mutations. Most HIDS patients are heterozygous for two different variants. The presence of homozygous I268T mutations is associated with MA, the most severe phenotype (42).

MA has a neonatal onset with repeated attacks of fever accompanied with severe ocular and neurologic involvement, musculoskeletal abnormalities associated with growth retardation and dysmorphic features, hepatosplenomegaly, lymphadenopathy, and cutaneous lesions (46). HIDS is clinically characterized by an early onset of monthly or bimonthly recurrent febrile attacks lasting from 3 to 7 days. Other typical features include cervical or generalized lymphadenopathies, prominent oral aphthae, arthralgia or non-erosive arthritis of large joints, abdominal pain, and hepatosplenomegaly. Attacks of systemic and cutaneous symptoms are occasionally triggered by infections, vaccines, or trauma (47).

Acute phase reactants, IgD and IgA levels are usually elevated during attacks. An increase of urinary mevalonic acid levels during attacks is considered a specific marker for MKD. Secondary amyloidosis has been found in about 3% of patients (47).

Glucocorticoids at high doses are useful to control attacks in some patients, but most of them will require biologic therapy to avoid glucocorticoid adverse events. Among biologics, etanercept may improve symptoms in more than 50% of patients. However, IL-1 blockers are effective in the majority of cases (37). Anakinra has been proved to be useful in continuous or on demand administration (48) and canakinumab has been recently approved by the FDA and the EMA for HIDS treatment (28). Tocilizumab has been reported effective in some cases refractory to previous treatments (49).

##### Dermatologic manifestations

Skin involvement occurs in about 70% of MKD patients (47). Cutaneous lesions are heterogeneous and typically consist of non-specific maculopapular or morbilliform rashes. Small erythematous macules, papules, nodules, or cellulitis-like plaques are also frequent. Erythema nodosum and urticarial lesions have also been described, as well as petechiae or purpura resembling IgA vasculitis, erythema elevatum diutinum, and Sweet's syndrome (16, 31, 32, 50). Bipolar aphthae are present in almost 50% of patients (47).

##### Cutaneous histopathology

MKD cutaneous lesions are histologically variable. Endothelial swelling and perivascular inflammatory infiltrate are the main changes in a skin biopsy. In addition, signs of leukocytoclastic or necrotizing vasculitis, Sweet-like lesions, erythema elevatum diutinum, or erythema nodosum may also be observed. Direct immunofluorescence may show perivascular and linear deposits of IgD and C3 along the basal membrane (14, 51).

#### Otulipenia

Otulipenia, also known as OTULIN-related autoinflammatory syndrome (ORAS), is an autosomal recessive autoinflammatory disease due to mutations in the *FAM105B* gene, which encodes OTULIN, a Met-1 specific deubiquitinase that acts as a negative regulator of the NF-κB signaling pathway (10).

Clinically these patients present with an early-onset of prolonged recurrent episodes of fever, erythematous skin rash with nodules, arthralgia, abdominal pain, diarrhea, lymphadenopathy, and elevated acute phase reactants (10).

Treatment with TNF inhibitors is very effective in controlling disease activity (10).

##### Dermatologic manifestations

A painful erythematous rash with skin nodules is the most frequent cutaneous manifestation. Other features include pustular rash, lipoatrophy, and panniculitis (10, 52).

##### Cutaneous histopathology

Skin biopsies show different types of panniculitis and neutrophilic dermatosis. Small and medium-sized vessel vasculitis have also been reported (10, 52).

#### HOIL-1 Deficiency

HOIL-1 deficiency is an autosomal recessive disease caused by mutations in the *HOIL1* gene, which encodes HOIL1, a component of the linear ubiquitination chain assembly complex (LUBAC). These mutations result in destabilization of LUBAC complex with an impairment of the IL-1β dependent NF-κB activation in fibroblasts. However, myeloid cells, in particular monocytes, are hyperreactive to IL-1β. Therefore, the consequences of human HOIL-1 and LUBAC deficiencies for IL-1β responses differ between cell types (10).

HOIL-1 deficiency is clinically characterized by an early-onset of recurrent episodes of fever with gastrointestinal symptoms, such as abdominal pain, vomiting, and diarrhea with blood and mucus, and also lymphadenopathy, respiratory distress, failure to thrive, and muscular amylopectinosis (storage of abnormal glycogen that leads to intracellular glycogen inclusions), which is complicated by myopathy and cardiomyopathy. Recurrent bacterial infections secondary to immunodeficiency features, including hyper-IgA syndrome and memory B-cell defects with antibody production deficiency and impaired response to vaccines have been reported. Inflammatory symptoms are accompanied by elevated acute phase reactants during flares (10, 53).

##### Dermatologic manifestations

Eczematous lesions, erythroderma, and exfoliative dermatitis occurred in different patients with HOIL-1 deficiency. Vaccination-induced subcutaneous inflammatory lesions have also been described (10, 53).

##### Cutaneous histopathology

No data regarding HOIL1 deficiency and cutaneous histology is available.

### Urticarial Rashes

#### Cryopyrin-Associated Periodic Syndromes (CAPS)

CAPS or cryopirinopathies comprise three autosomal dominant conditions with different disease severity. The mildest form is familial cold autoinflammatory syndrome (FCAS), the intermediate phenotype is Muckle-Wells syndrome (MWS), and the most severe form is neonatal-onset multisystem inflammatory disease (NOMID), also known as chronic infantile, neurologic, cutaneous and articular (CINCA) (54). All CAPS are caused by mutations in the *NLRP3* gene, which encodes NLRP3 protein or cryopyrin and lead to constitutive activation of NLRP3 inflammasome and IL-1β overproduction. However, more than half of CINCA/NOMID cases are produced by *de novo* mutations. While the presence of pathogenic mutations predicts a more severe phenotype with neurologic complications and sensorineural hearing loss, low penetrance or uncertain significance variants are associated with milder disease phenotypes (11, 54).

Common clinical features to all CAPS forms include an early disease onset with fever or low-grade fever episodes, fatigue, urticarial rash, musculoskeletal symptoms, and ocular involvement as conjunctivitis and uveitis. During attacks, acute phase reactants tend to be elevated (14, 17). In FCAS, attacks are typically triggered by cold exposure and self-limited in <24 h. In MWS, attacks usually last 1–2 days and sensorineural hearing loss and amyloidosis are frequently developed, mostly in undiagnosed or untreated patients. CINCA/NOMID is characterized by a sustained systemic inflammatory response that included persistent fever, diffuse urticarial lesions and severe osteoarticular, ocular and neurologic involvement, usually leading to deforming and irreversible sequelae. Without a prompt directed treatment, CINCA/NOMID becomes a disabling and lethal disease.

Anti-IL-1 agents are considered the treatment of choice for CAPS (39, 55, 56) since anakinra and canakinumab are approved by the FDA and the EMA for CAPS treatment. While IL-1 blockade does not appear to influence established joint and bone damage, its early administration seems to reduce the risk of developing (or improve them when developed) amyloidosis, hearing loss, and neurologic complications (57).

##### Dermatologic manifestations

**Figure 3:**
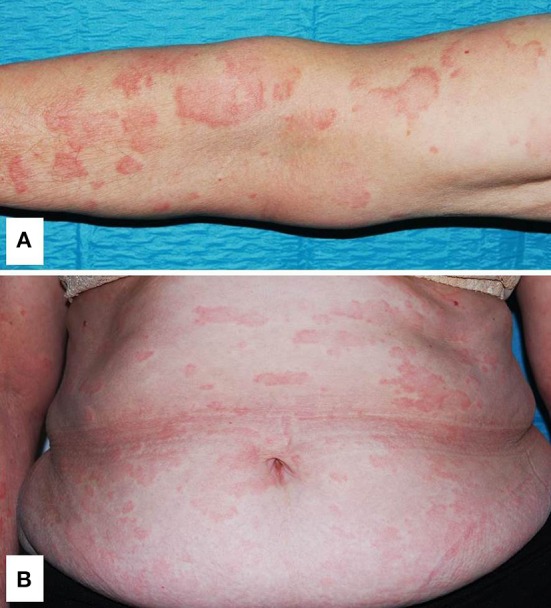
Generalized urticarial rash with erythematous flat wheals without surrounding flare on the left arm **(A)** and trunk **(B)** in a patient with Muckle-Wells syndrome. Written informed consent was obtained from the patient for the publication of this image.

A non-pruritic, somewhat symmetrical and evanescent urticarial rash involving the trunk and extremities, usually sparing the head, is the most frequent cutaneous event in CAPS (Figure 3) (20, 31, 58). As for CAPS, in other monogenic autoinflammatory diseases with urticarial lesions, hives are usually more flattened, painful or burning, and last longer than those of chronic spontaneous urticaria. In addition, they may also appear as erythematous patches or even solid lesions. Angioedema is not usually present. Although FCAS attacks are usually triggered by cold exposure, contact with cold objects does not cause a disease attack, and therefore, the ice cube test is negative (20).

##### Cutaneous histopathology

**Figure 4:**
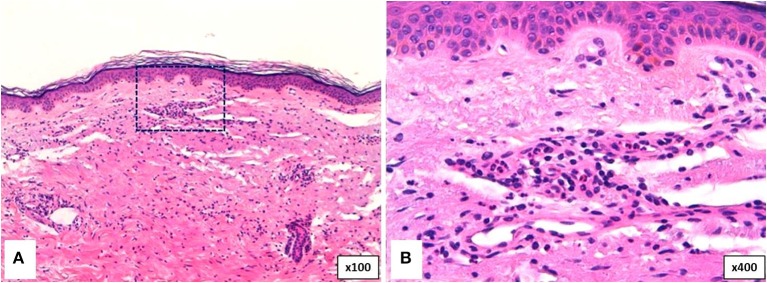
Histopathology of a wheal from a patient with Muckle-Wells syndrome (MWS). **(A)** Dermal interstitial and perivascular infiltrates composed of lymphocytes and neutrophils consistent with neutrophilic urticaria. **(B)** Perivascular infiltrate in detail.

**Figure 5:**
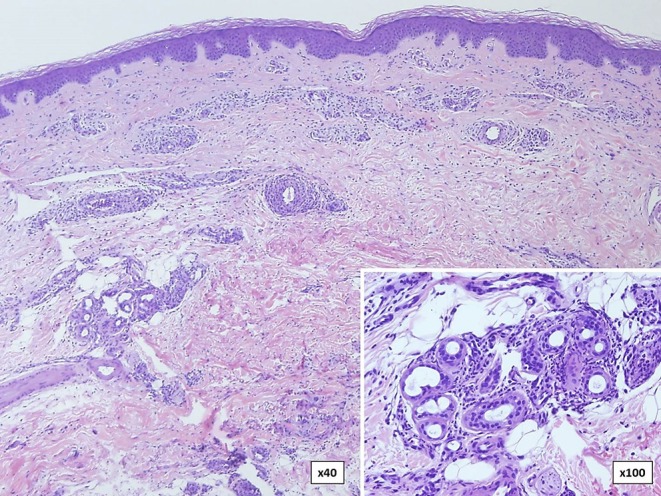
Skin biopsy in a patient with familial cold autoinflammatory syndrome (FCAS) due to a somatic mutation in the *NLRP3* gene. There are dermal neutrophilic infiltrates between the collagen bundles and around blood vessels with presence (inset) of neutrophils around and within eccrine glands.

Neutrophilic urticarial dermatosis is the clinicopathological term used to describe dermatologic and histological findings in CAPS, which are different from those observed in ordinary neutrophilic urticaria. CAPS skin biopsies usually show no edema or mild dermal edema of the papillary dermis with a perivascular and neutrophilic infiltrates with limited leukocytoclasia (32) (Figure 4). The presence of neutrophilic epitheliotropism (neutrophils around or within eccrine glands or ducts, or inside the epidermis) is rather characteristic although it can be seen in other entities (Figure 5) (59). Interstitial neutrophilic infiltrates have also been described (14, 60, 61).

#### NLRP12-Associated Autoinflammatory Disease (NLRP12-AD)

NLRP12-AD, also known as FCAS2, is an autosomal dominant autoinflammatory disease caused by mutations in the *NLRP12* gene, which encodes Monarch-1 (31) and play a role in the activation of NF-κB and caspase 1 signaling pathways (62).

Similarly, to FCAS, patients with NLRP12-AD present with recurrent episodes of high fever triggered by cold exposure, lasting for 2–10 days, every 3–4 weeks. Fever is commonly accompanied by arthralgia, myalgia, abdominal pain, headache, lymphadenopathy, oral aphthae, and skin rash. Sensorineural hearing loss is the most common long-term complication. Acute phase reactants are elevated during attacks (63, 64).

Glucocorticoids, antihistamines, and NSAIDs may be useful in mild cases. Severe cases seem to respond to anakinra and also to anti-IL-6 and anti-TNFα agents (64, 65).

##### Dermatologic manifestations

Cold exposure usually induces the attack and cutaneous manifestations consisting of an evanescent urticarial rash involving the trunk, extremities and face. An erythematous malar rash (64) and cutis laxa (66) have also been described to occur. Contrary to FCAS, FCAS2 rashes tend to be itchy. The ice cube test is consistently negative (63, 64).

##### Cutaneous histopathology

No data about NLRP12-AD cutaneous histology is available. However, histopathological findings are expected to be similar to those described in CAPS.

#### PLCγ2-Associated Antibody Deficiency and Immune Dysregulation (PLAID)

PLAID, also known as FCAS3, is an autosomal dominant autoinflammatory disease due to mutations in the *PLC*γ*2* gene, encoding phospholipase Cγ2 (PLCγ2), a transmembrane signaling enzyme with phospholipase activity. Cellular dysregulation is produced by a signaling reduction on pathways depending of PLCγ2, which are enhanced at low temperatures. B cells, NK cells, and mast cells are involved in the inflammatory dysregulation (67). *De novo* mutations have been also reported (68).

Clinical manifestations include an early onset of recurrent cutaneous lesions triggered by cold exposure and immunological abnormalities, such as the presence of antinuclear antibodies (ANA), immunoglobulin deficiencies (mostly IgM and IgA), elevated IgE levels, and decreased amounts of switched memory B-cells resembling a primary immunodeficiency, which leads to an increased susceptibility to infections (68).

Avoiding cold temperatures is the main preventive therapy. Depending on the history of repeated infections, intravenous immunoglobulins and prophylactic antibiotics can be used (69). Directed therapies with PLCγ2 inhibitors are not available yet (70).

##### Dermatologic manifestations

The main cutaneous manifestation of PLAID is a recurrent itchy cold-induced evaporative urticaria, since it appears in cold-sensitive regions of the body after generalized exposure to cold air or evaporative cooling, but not after contact with cold objects. Lesions subside with an increase in temperature (69, 70). Other less common features include a neonatal ulceration of the nasal tip, which may show spontaneous regression or have a progressive and destructive course, and small papules and erosions on the fingers and toes that tend to resolve without sequelae. Granulomatous-like inflammatory lesions, usually presenting as red-brown, indurated and scaly plaques and nodules of the skin sparing warm regions, such as flexural surfaces and skinfolds (69), and infantile epidermolysis-bullosa-like eruption, initially generalized and later evolving to recurrent erythematous plaques and vesiculopustular photosensitive lesions (71) have also been reported.

##### Cutaneous histopathology

Urticarial lesions show an increased number of perivascular and interstitial mast cells, which appear degranulated after cold exposure (72). Biopsies of granulomatous lesions reveal well-delineated, non-necrotizing, non-caseating, or sarcoid-type granulomas, but also diffuse, poorly-defined granulomatous inflammation, particularly in the superficial dermis. Granulomatous infiltrates are composed by nodular foci of CD68+ epithelial histiocytes and multinucleated giant cells surrounded by a mild CD4/CD8+ lymphocytic infiltrate and scattered eosinophils. Perineural and lymph nodes granulomatous inflammation may also be observed (69).

#### NLRC4-Associated Autoinflammatory Diseases (NLRC4-AD)

NLRC4-associated macrophage activation syndrome (NLRC4-MAS) and familial cold autoinflammatory syndrome 4 (FCAS4) are part of NLRC4-AD (73). Both phenotypes are autosomal dominant diseases caused by mutations in the *NLRC4* gene, encoding NLRC4, which lead to a constitutive NLRC4 inflammasome activation resulting in an increased secretion of IL-1β and IL-18. IL-18 is found at extremely high levels in patients with NLRC4-MAS and may persist elevated, even in the absence of clinical activity (74, 75).

The most severe clinical phenotype (NLRC4-MAS) is dominated by a multisystemic inflammation starting in the first year of life with symptoms of chronic inflammatory bowel disease, MAS, or symptoms resembling CINCA/NOMID. Enterocolitis tends to subside over time (74). The mildest phenotype (FCAS4) usually starts at age of three with attacks after exposure to cold stimuli of urticaria, arthralgia, ocular inflammation, and fever in half of cases, in absence of visceral involvement. Although CRP levels are elevated, in severe cases, ESR values tend to decrease as the disease progresses.

Glucocorticoids and anakinra may be useful in most mild cases (76). IL-18 inhibitors and anti-interferon-gamma inhibitors have shown good response in severe cases (73, 75).

##### Dermatologic manifestations

Skin manifestations range from an unspecific rash to cold urticaria, evanescent urticarial, or linear erythematous lesions (74). While children commonly present with urticarial rash alone, in adult patients, urticarial lesions, and painful erythematous nodules on lower extremities are the most frequent signs (77, 78).

##### Cutaneous histopathology

NLRC4-AD histopathological findings are scarce. Nodular lesions show deep dermal and subcutaneous lymphohistiocytic infiltrates with septal and lobular panniculitis. Perivascular lymphocytic infiltrates without vasculitic changes have also been described. Direct immunofluorescence has not detected IL-1β staining (77).

#### Vibratory Urticaria

Vibratory urticaria is an autosomal dominant autoinflammatory disease caused by mutations in the *ADGRE2* gene, which encodes ADGRE2, a member of the epidermal growth factor seven transmembrane that acts as a cell surface receptor with two subunits, the extracellular α subunit and the transmembrane β subunit. It is predominantly expressed in leukocytes, especially in neutrophils and macrophages, but also in mast cells. The endogenous ligand of ADGRE2 is dermatan sulfate, which is the predominant glycosaminoglycan of the skin. The mutated ADGRE2 receptor undergoes autocatalytic cleavage, producing an extracellular subunit that non-covalently binds a transmembrane subunit with destabilization of the autoinhibitory subunit interaction and sensitization of mast cells to IgE-independent vibration-induced degranulation. Therefore, transitory high histamine serum levels seem to be responsible for the clinical manifestations in these patients (79).

Localized pruritic hives after repetitive vibratory or friction stimuli are the principal manifestations of the disease. Occasionally, cutaneous lesions may be accompanied by systemic symptoms (79).

##### Dermatologic manifestations

Skin lesions consist of localized pruritic hives, angioedema, erythema, and pruritus caused by repetitive physical stimulation. Cutaneous changes may appear from a few minutes to an hour after the vibratory stimulus. In prolonged or intense mechanical expositions, urticarial lesions may be associated with a more severe angioedema or systemic symptoms, such as headache, fatigue, facial flushing, and metallic taste. While dermographism is not present in patients with vibratory urticaria, urticarial rash can be provoked by stimulating the forearm with a laboratory vortex (79).

##### Cutaneous histopathology

Skin biopsies of vibration-induced lesions show a significant release of mast cell granular content in cases samples compared to controls (79).

### Pustular, Pyogenic, or Neutrophilic Dermatosis-Like Rashes

#### Pyogenic Arthritis, Pyoderma Gangrenosum, and Acne Syndrome (PAPA)

PAPA is an autosomal dominant autoinflammatory disease caused by mutations in the *PSTPIP1* gene, encoding CD2BP1 or PSTPIP1 (80). Although the pathogenesis is not completely understood, PSTPIP1 seems to play a role in inflammasome activation and overproduction of IL-1β and IL-18 (81).

Clinical manifestations start at pediatric age with recurrent flares of erosive, sterile, and deforming arthritis of the elbows, ankles, and knees, leading to early joint destruction. Skin ulcers and severe acne occur during adolescence. Fever is rare and frequency of flares tends to decrease with age. Increased acute phase reactants and leukocytosis are observed during attacks (82).

Glucocorticoids, IL-1 blockers, and anti-TNF agents may be useful in treating arthritis and pyoderma gangrenosum (83).

After PAPA description in 1997 by Lindor et al. (84), other pyoderma gangrenosum-associated syndromes (85) with autoinflammatory background and a late-onset have been described. Those include PASH (86–89), PAPASH (83, 90), PsAPASH (91), and PASS (92). These PAPA-like syndromes are summarized in Table 3.

**Table 3:** Characteristics of the pyoderma gangrenosum-associated autoinflammatory syndromes.

	**PASH syndrome (86–89)**	**PAPASH syndrome (83, 90)**	**PsAPASH syndrome (91)**	**PASS syndrome (92)**
Complete name/Clinical manifestations	Pyoderma gangrenosum, acne, and hidradenitis suppurativa	Pyogenic/psoriasis arthritis, pyoderma gangrenosum, acne, hidradenitis suppurativa	Psoriatic arthritis, pyoderma gangrenosum, acne, hidradenitis suppurativa	Pyoderma gangrenosum, acne, hidradenitis suppurativa, seropositive spondyloarthropathy
Year of description	2012	2013	2015	2012
Mutated genes	*NCSTN, PSMB8, NOD2, MEFV, IL1RN, NLRP3, PSTIP1*	*PSTPIP1* (E277D)	Unknown	Unknown
Treatment reported	Dapsone, cyclosporine, IL-1 blockers, infliximab, adalimumab	Glucocorticoids, cyclosporine, anakinra, adalimumab, infliximab, secukinumab	Glucocorticoids, cyclosporine, anakinra, adalimumab, infliximab	Infliximab

##### Dermatologic manifestations

**Figure 6:**
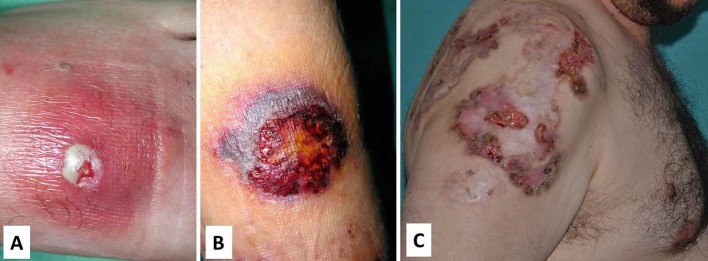
Different stages of pyoderma gangrenosum in PAPA. **(A)** Initial lesion with an erythematous and tender plaque with a central sterile pustule; **(B)** Ulceration with necrotic borders; and **(C)** Poor-healing and painful ulcer with undermined borders and cribriform scarring. Written informed consent was obtained from the patients for the publication of these images.

Skin involvement includes pyoderma gangrenosum and severe cystic acne, which gets worse with puberty. Pyoderma gangrenosum may occur spontaneously or be triggered by trauma (pathergy) and starts as a violaceous tender papule, nodule or a sterile pustule that rapidly expands with necrosis of the surrounding tissue, and finally results in a poor-healing and painful ulcer with undermined borders (Figure 6). Granulation tissue, necrosis or purulent discharge is common in the middle of the ulcer. Cribriform scarring is a hallmark of the disease and may help with the diagnostic (93). Psoriasiform lesions and rosacea-like eruptions have also been reported (94).

##### Cutaneous histopathology

**Figure 7:**
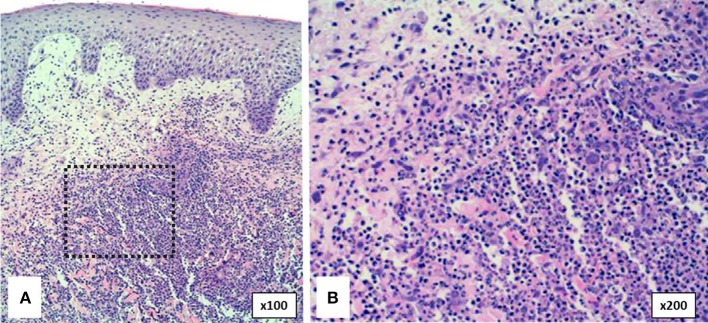
Histopathology of pyoderma gangrenosum in a PAPA patient. **(A)** Dense neutrophilic infiltrate with upper dermis edema. **(B)** Neutrophilic infiltrate in detail.

The typical histological feature consists of central sterile neutrophilic infiltrates in the dermis that becomes with mixed cellularity in the peripheral areas (Figure 7) (14, 93).

#### Deficiency of IL-1 Receptor Antagonist (DIRA)

DIRA is an autosomal recessive autoinflammatory disease caused by mutations in the *IL1RN* gene, encoding IL-1 receptor antagonist (IL-1RA) (95). This mutations lead to the absence of IL-1RA and produce an overactivity of IL-1 (57).

DIRA is clinically characterized by a neonatal-onset of chronic-recurrent flares with cutaneous pustulosis, joint swelling, and bone pain due to painful multifocal aseptic osteomyelitis, long bone periostitis, epiphyseal overgrowth, and secondary skeletal malformations. Interstitial lung disease, vasculitis of the central nervous system, thrombosis, and respiratory distress are much less frequent manifestations (96, 97). Although fever is usually absent, acute phase reactants are constantly elevated during attacks. If untreated, the disease tends to evolve to multiorgan failure with a high mortality rate (57, 96).

Anakinra at doses of 1–5 mg/kg/day remains the treatment of choice for DIRA since it produces a fast and complete clinical and biological resolution in the majority of patients (98).

##### Dermatologic manifestations

Newborn children present with localized or generalized erythematous plaques and overlying sterile pustules sparing palms and soles. These plaques may evolve to diffuse desquamation resembling ichthyosiform lesions. Nail changes with pitting and onychomadesis, similar to those experiencing in psoriasis, are frequent. Oral lesions such as ulcers and vesicular stomatitis may also occur (97).

##### Cutaneous histopathology

Histological findings resemble those of pustular psoriasis and skin biopsies show acanthosis and hyperkeratosis of the epidermis with extensive epidermal and dermal neutrophilic infiltrates developing pustules around hair shafts. Vasculitis in subcutaneous tissue adjacent to the bone have also been described (96, 99).

#### Deficiency of IL-36 Receptor Antagonist (DITRA)

DITRA is an autosomal recessive autoinflammatory disease caused by mutations in the *IL36RN* gene, which encodes IL-36 receptor antagonist (IL36Ra) (100). These mutations are involved in NF-κB activation and overproduction of proinflammatory cytokines such as IL-36 and IL-8 (100, 101). In recent years, late-onset cases have been described in patients carrying heterozygous mutations (101).

DITRA is clinically included in generalized pustular psoriasis. These patients may have a pediatric and adult onset consisting of irregular episodes of high-grade fever, generalized pustulosis, and asthenia, with elevated acute phase reactants and leukocytosis. Attacks have been reported to be triggered by infections, pregnancy, and menstruation (100). Several authors have suggested that patients with early-onset generalized pustular psoriasis without concomitant psoriasis vulgaris are often diagnosed with DITRA (102).

DITRA is currently included in the group of autoinflammatory keratinization diseases (AIKD), a term first used in 2017 to cluster those disorders characterized by keratinized lesions caused by an autoinflammatory mechanism (103).

Conventional topical and systemic therapies used for psoriasis may also be useful in DITRA patients (16). Anakinra (anti-IL-1), adalimumab, infliximab (anti-TNFα), ustekinumab (anti-IL-12/23), and secukinumab (anti-IL-17) have shown efficacy in isolated cases (104–106). Recently, a phase 1 clinical trial in patients with generalized pustular psoriasis treated with a single intravenous dose of a monoclonal antibody against the IL-36 receptor has shown promising results by reducing the severity of the disease over a 20-week period, regardless of the presence of the *IL36RN* mutation (107).

##### Dermatologic manifestations

**Figure 8:**
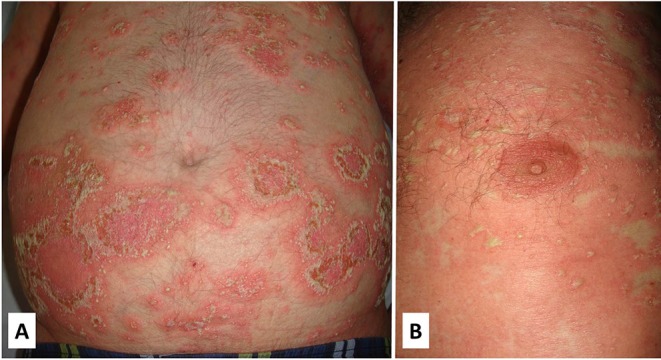
Clinical features of a patient with DITRA with a heterozygous mutation in the *IL36RN* gene. The clinical picture started in adulthood with flares of diffuse erythematous plaques covered with pustules that often involved the whole body **(A,B)**. The episodes were often triggered by bacterial infections. Written informed consent was obtained from the patient for the publication of this image.

Cutaneous lesions resemble those of generalized pustular psoriasis and consist of a diffuse erythematous skin eruption that tends to be rapidly covered by pustules with subsequent desquamation (Figure 8). Skin eruptions may mimic all forms of psoriasis ranging from psoriasis vulgaris to acrodermatitis continua (100, 108).

##### Cutaneous histopathology

**Figure 9:**
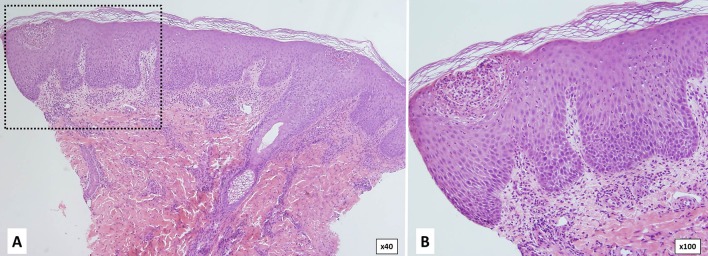
Histology of DITRA shows epidermal acantosis with edema and dilated blood vessels in the papillary dermis. There is epidermal spongiosis with the presence of neutrophils migrating through the epidermis **(A,B)**. In the most superficial part of the epidermis (inset-**B**) there is a subcorneal pustule that is formed through the aggregation of neutrophilic spongiform pustules.

Histological features are indistinguishable from classical pustular psoriasis and include epidermal hyperplasia with acanthosis, irregular papillomatosis, subcorneal spongiform pustules, compact orthokeratosis, or parakeratosis and neutrophilic infiltration (Figure 9). Immunohistochemistry of the dermis shows superficial perivascular infiltrates of CD8 and CD3 T cells, macrophages and neutrophils (100, 108).

#### CARD-14 Mediated Psoriasis (CAMPS)

CAMPS is an autosomal dominant inherited disease due to mutations in the *CARD14* gene encoding CARD14. Such mutations produce an overactivation of NF-κB pathway (109). Keratinocytes show high levels of CARD14 (110). CAMPS is currently classified as AIKD (103).

Disease presentation may vary among monogenic psoriasis, pustular psoriasis, psoriatic arthritis, or pityriasis rubra pilaris. Features of systemic inflammation are usually absent. Therapeutic options are mainly the same as those used for treating psoriasis and DITRA (111–113).

##### Dermatologic manifestations

Clinical manifestations are mostly cutaneous presenting as a plaque and pustular psoriasis. Other diseases, such as pityriasis rubra pilaris or acute generalized exanthematous pustulosis, have also been associated with CARD14 mutations. Disease extension may vary from localized to generalized, as well as severity, which may range from mild to severe (111, 113–115).

##### Cutaneous histopathology

Skin biopsies show histopathological features of psoriasis or pityriasis rubra pilaris (116).

#### Majeed Syndrome

Majeed syndrome is an autosomal recessive autoinflammatory disease caused by mutations in the *LPIN2* gene, which encodes phosphatase lpin2 (117). Mutated LPIN-2 induces NLRP3 activation with the consequent IL-1β overproduction (118).

The clinical triad is characterized by the early onset of chronic recurrent multifocal sterile osteomyelitis (CRMO), congenital dyserythropoietic anemia and neutrophilic skin lesions (119). Other manifestations during attacks include fever and swelling of large joints. Growth retardation and permanent flexion contractures are long-term complications in untreated patients (120). Abnormal laboratory tests include raised acute phase reactants levels, anemia, and variable leukocytosis.

Treatment with NSAIDs and glucocorticoids may be useful in controlling CRMO-related pain. Anti-TNF agents, bisphosphonates, and interferon gamma show variable success rates. IL-1 blockers have been useful in controlling inflammatory manifestations (121–123).

##### Dermatologic manifestations

Inflammatory dermatoses are the most frequent cutaneous symptoms, and may occur as neutrophilic dermatoses and erythematous and scaly plaques. The prototypic findings are Sweet syndrome-like lesions, seen as erythematous plaques, pseudovesiculous or target lesions (120, 124). CRMO has also been associated with generalized pustulosis, palmoplantar psoriasis, pyoderma gangrenosum, acne, recurrent subcutaneous abscesses, and SAPHO syndrome (31, 120, 125–128).

##### Cutaneous histopathology

Skin histopathology displays edema of the papillary dermis with dense dermic neutrophilic infiltrates. A bone biopsy usually shows subacute and chronic inflammatory changes (129).

### Pyrin-Associated Autoinflammation With Neutrophilic Dermatosis (PAAND)

PAAND is an autoinflammatory disease caused by mutations in the *MEFV* gene, the same gene responsible for FMF. However, contrarily to the autosomal recessive but inconstant pattern observed in FMF patients, PAAND has an autosomal dominant inheritance with complete penetrance. PAAND mutations (S242R and E244K) are associated with pyrin inflammasome activation (130).

PAAND has a childhood-onset characterized by recurrent febrile episodes lasting for several weeks accompanied with arthralgia, myalgia and cutaneous inflammatory lesions. During attacks, acute phase reactants and circulating proinflammatory cytokines (IL-1β, IL-6, TNF-α, and IL-1Ra) levels are normally increased (130).

Treatment with IL-1 blockers has shown a rapid control of clinical and laboratory abnormalities. Infliximab and adalimumab have been used with success in anakinra-resistant patients (130).

#### Dermatologic manifestations

Severe neutrophilic dermatoses in PAAND have a wide spectrum of presentation, including pustular acne, pyoderma gangrenosum, sterile skin abscesses, neutrophilic small vessel vasculitis, severe hidradenitis suppurativa, and neutrophilic panniculitis (32, 130, 131).

#### Cutaneous histopathology

Histopathology reveals an spared epidermis and dense dermal neutrophilic infiltrates both interstitial and perivascular (131).

#### Other Novel Psoriasiform Monogenic Autoinflammatory Diseases

##### Singleton-merten syndrome (SMS)

SMS is an autosomal dominant transmitted disease caused by mutations in *IFIH1* or *DDX58* genes. The resulting proteins (melanoma differentiation associated protein 5 [MDA5] and retinoic-acid-inducible gene I [RIG-I], respectively) are involved in type I interferon induction pathways (132).

Clinical manifestations occur after childbirth and are characterized by dental dysplasia, tendon rupture, osteoporosis, arthropathy, neurologic abnormalities, aortic calcification, and glaucoma. Cutaneous involvement as localized or generalized psoriasis is present in the majority of patients (132).

As in other type I interferonopathies, the use of a Janus kinase (JAK) inhibitor has been useful in a SMS patient (132).

##### ADAM17 deficiency

ADAM17 deficiency is considered an autoinflammatory disease (6) caused by autosomal recessive mutations in the *ADAM17* gene, encoding TNF-α converting enzyme (TACE), which is necessary for the cleavage and secretion of TNF-α, epidermal growth factor, transforming growth factor alpha (TGF-α), and some desmogleins (6, 133).

Clinical features were described in two consanguineous siblings with neonatal-onset of pustular psoriasis followed by chronic bloody diarrhea and cardiomyopathy. Skin lesions were characterized by perioral and perianal erythema with fissuring and a generalized pustular rash that evolved to psoriasiform erythroderma, with flares of erythema, scaling, and widespread pustules. Cutaneous infections were frequent. Other dermatologic manifestations included hair abnormalities (short or fragile hair and wiry eyelashes and eyebrows), and thickened nails with frequent episodes of paronychia. Dermatopathology revealed infiltrates of T cells in the epidermis. CD3+ T cells were located around the skin follicles and in the epithelium, CD4+ T cells in the perifollicular region and CD8+ T cells in the epithelium at the neck of the follicle. B cells (CD20+), natural killer cells (CD56+), or neutrophils were scarce within the infiltrates (133).

Treatment with acitretin, ciclosporin, methotrexate, and adalimumab has not been useful in patients with ADAM17 deficiency. However, anti-IL1 and anti-IL6 therapy may be potential agents since peripheral-blood mononuclear cells from patients overproduced IL-1β and IL-6 after lipopolysaccharide stimulation (133).

##### AP1S3 and autoinflammatory psoriasis

Pustular psoriasis may be caused by mutations in the *AP1S3* gene encoding AP1S3, a protein implicated in autophagosome formation, which is elevated in keratinocytes. Its deficiency disrupts keratinocyte autophagy and causes abnormal accumulation of p62, an adaptor protein mediating NF-kB activation, with subsequent up-regulation of IL-1 signaling and overexpression of IL-36. The inheritance pattern is not clear since patients with *de novo* mutations and with a mutated allele from an unaffected parent have been reported. Treatment with IL-36 blockade has demonstrated to reverse skin lesions (134).

Although inflammatory symptoms such as arthritis may be present, pustular psoriasis is the most prominent clinical feature. This may be localized to the palms and soles (palmar plantar pustulosis) or to the toes and fingertips (acrodermatitis continua of Hallopeau), but it may also be generalized (134).

### Panniculitis or Subcutaneous Nodules

#### Blau Syndrome and Early-Onset Sarcoidosis

Blau syndrome and early-onset sarcoidosis are the two forms of pediatric granulomatous arthritis caused by mutations in the *NOD2*/*CARD15* gene encoding NOD2. While Blau syndrome is inherited in an autosomal dominant manner, early-onset sarcoidosis is the spontaneous form, caused by the novo mutations (135).

In both disorders, symptoms onset occurs during the first decade of life with the sequential, but not constant, triad of maculopapular rash, non-erosive arthritis of wrists, hands, elbows and ankles, and uveitis. Other less frequent manifestations include fever, large and small vessel vasculitis, interstitial lung disease, cranial neuropathies, and granulomatous involvement of salivary glands, kidneys, spleen, and liver (136, 137). Laboratory studies are typically normal, although elevated ESR and angiotensin-converting enzyme levels and hypergammaglobulinemia have been reported (137).

With regard to treatment, high-dose glucocorticoids may be useful for inflammatory symptoms. Limited reports have shown effectiveness with thalidomide, methotrexate, cyclosporine and other conventional immunosuppressants, together with anti-IL-1 and anti-IL-6 agents. However, anti-TNF blockers (infliximab and adalimumab) seem to be the drugs associated with better responses (15, 135–137).

##### Dermatologic manifestations

Skin involvement is the most prominent and the earliest expression of the disease, which is manifested as an erythematous maculopapular fine scaly rash on the trunk and extremities, resembling an ichthyosiform exanthema. Progressively it becomes tan-colored with lichenoid characteristics and dirty scaly appearance. This later stage tends to last longer (22, 138). Erythema nodosum-like lesions, pityriasis lichenoides, leg ulcers, and leukocytoclastic vasculitis have also been observed (136, 139).

##### Cutaneous histopathology

Histopathology of the cutaneous lesions shows non-caseating, sarcoid-type granulomas in the subpapilar dermis with a variable number of lymphocytes and eosinophils (138). Biopsies from purpuric lesions display vasculitis, and leg ulcers can show both, granulomatous infiltrates and chronic granulation with mononuclear infiltration in the fat tissue (139).

#### Chronic Atypical Neutrophilic Dermatitis With Lipodystrophy and Elevated Temperature Syndrome (CANDLE)

CANDLE syndrome, also called proteasome associated autoinflammatory syndrome (PRAAS), is an autosomal recessive autoinflammatory disease caused by mutations in the *PSMB8* gene, which encodes the β5i subunit of the immunoproteasome (5, 11). *PSMB*9, *PSMA3, PSMB4*, and *POMP* are other proteasome genes recently identified as also causing CANDLE/PRAAS (140). This condition is considered an interferonopathy since mutant genes cause defective proteasome/immunoproteasome assembly and accumulation of ubiquitinated proteins that induce intracellular stress and increased IFN-1 production through JAK signaling pathway (141, 142).

Classical manifestations include neonatal onset of recurrent or persistent high-fever, cutaneous lesions, and facial and generalized lipodystrophy. Arthralgia, muscle atrophy, hepatosplenomegaly, lymphadenopathy, and inflammatory involvement of other territories, such as ocular, meningeal, epididymis and parotids, are also common (143). Raised acute phase reactants are constant and muscle and hepatic enzymes are frequently elevated. Positive ANA and antineutrophil cytoplasmic antibodies (ANCA) may be present without pathogenic significance (144).

Glucocorticoids, conventional immunosuppressive drugs, and biologic agents, such as anti-TNF, anti-IL-1, or anti-IL-6 have been used without complete response (143, 144). Baricitinib, a JAK inhibitor that prevents the expression of IFN-induced genes and the autoinflammatory loop, has shown efficacy in CANDLE/PRAAS patients (145).

##### Dermatologic manifestations

Perinatal-onset fever attacks are accompanied by annular erythemato-violaceous edematous plaques on trunk and extremities, and stable violaceous erythemas on the perioral and periorbital areas. Most of these lesions resolve within few days or weeks leaving purpuric pigmentation, but recurrences are common. Other less frequent manifestations include violaceous nodules, hirsutism, and acanthosis nigricans. The development of progressive lipoatrophy of the face, extremities, and trunk occurs in the late phase of the disease (22, 143, 144).

##### Cutaneous histopathology

Histopathology of cutaneous lesions is characterized by dense interstitial and perivascular atypical-looking (because of the presence of mitotic figures) mononuclear infiltrates with karyorrhexis in the deep dermis and fat tissue. Neutrophils and eosinophils may also be observed within the infiltrates. Immunohistochemistry shows strong and diffuse positivity for myeloperoxidase, lysozyme, CD68, and CD45, which confirms the myeloid lineage of the infiltrate by revealing the presence of macrophages and histiocytes. T cells and B cells, identified by positivity for CD3, CD45RO, and CD20, are also present to a lesser extent (143, 144).

### Vasculitis or Vasculopathy

#### Deficiency of Adenosine Deaminase 2 (DADA2)

DADA2 is an autosomal recessive autoinflammatory disease caused by mutations in the *CECR1* gene, encoding ADA2 (146, 147). ADA2 acts as a growth factor in the myeloid lineage promoting differentiation into anti-inflammatory macrophages, and has also a role in the development and maintenance of endothelial cells. Mutant ADA2 promotes vascular damage by affecting endothelial cells and inducing neutrophil-driven cell damage (146, 147).

DADA2 patients commonly exhibit persistent or recurrent fever, skin lesions (mostly livedo reticularis or racemosa and subcutaneous nodules), peripheral neuropathy and vascular lesions secondary to distal ischemia or hemorrhage of the affected territories, especially involving the brain. Disease phenotype is frequently indistinguishable from polyarteritis nodosa. Oral aphthae, arthralgia and hepatosplenomegaly are also frequent. Acute phase reactants are increased during attacks and the presence of variable peripheral blood cytopenias and low immunoglobulin levels contribute to develop a certain degree of immunodeficiency. Although disease typically occurs in early childhood, later-onset cases have also been described (146, 147).

Although high-dose glucocorticoids can be effective in some patients, low-dose glucocorticoids, conventional immunosuppressive drugs, anti-CD20 therapy and anti-IL-1 and anti-IL6 blockers do not seem to provide a clear benefit. However, anti-TNF agents, in particular etanercept, have demonstrated to control systemic inflammatory manifestations and progression of vascular disease, in the absence of normalization of ADA2 enzyme activity. To date, allogeneic hematopoietic stem cell transplantation is the only therapy that has demonstrated to cure the disease (146–148).

##### Dermatologic manifestations

**Figure 10:**
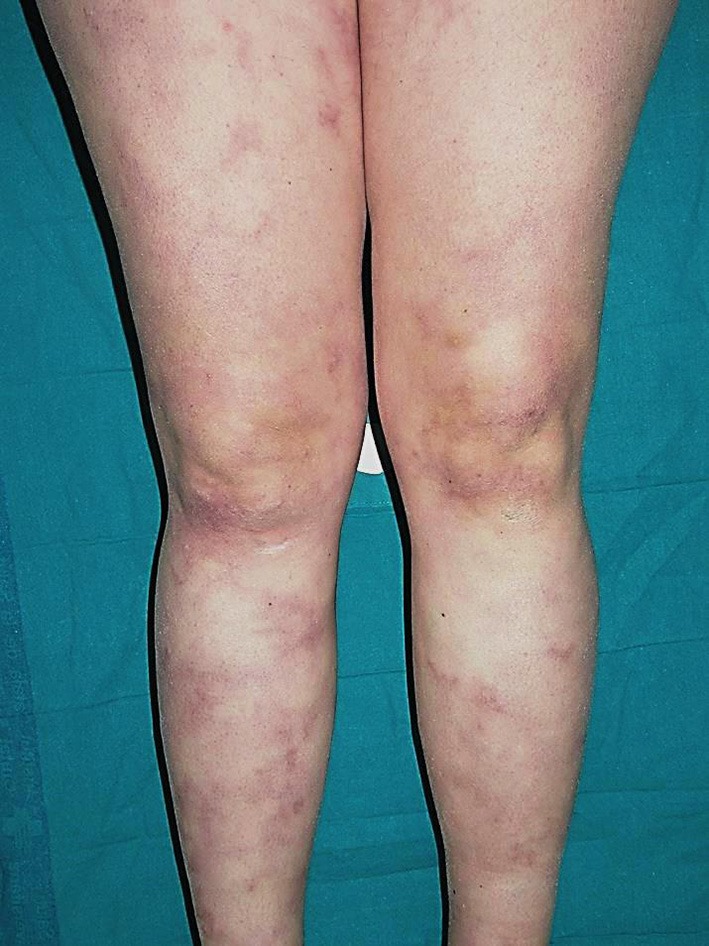
Livedo racemosa on the lower limbs in a patient with DADA2. Written informed consent was obtained from the patient for the publication of this image.

The most frequent cutaneous lesions are livedo reticularis or racemosa (Figure 10) and subcutaneous nodules. Pediatric and adult presentations may have a phenotype resembling cutaneous arteritis or polyarteritis nodosa refractory to conventional immunosuppressive therapy. Raynaud syndrome, digital necrosis, ulcers, and erythema nodosum may also occur (146, 147, 149).

##### Cutaneous histopathology

Skin biopsies are characterized by dermal interstitial neutrophilic infiltrates which stain positive for myeloperoxidase and CD68 confirming the existence of macrophages and a perivascular lymphocytic infiltrate. Livedo and nodular lesions may display non-granulomatous necrotizing medium-sized vessels vasculitis. However, leukocytoclastic vasculitis or panniculitis have also been reported (32, 146, 147, 149).

#### STING-Associated Vasculopathy With Onset in Infancy (SAVI)

SAVI is an autosomal dominant autoinflammatory disease caused by mutations in the *TMEM173* gene encoding STING, an indirect sensor of cytosolic DNA that activates IRF3 and induces transcription of IFN-1 related genes. Mutant STING results in overactivation of IRF3 and transcription of IFNβ (150).

SAVI is clinically characterized by a neonatal-onset of recurrent febrile attacks with cutaneous rash, small-vessel vasculitis, and interstitial lung disease. During flares, acute phase reactants are elevated and low-titer autoantibodies, such as ANA, ANCA, and antiphospholipid antibodies, are frequent (150, 151).

As in other interferonopathies, IFN-1 pathway blockade with JAK inhibitors, in particular baricitinib, seems to be effective in SAVI, since glucocorticoids, conventional immunosuppressive, and anti-cytokines agents have not demonstrated efficacy (145).

##### Dermatologic manifestations

Skin is the initial territory involved in SAVI. Lesions are caused by vasculitic changes with subsequent tissue damage and are manifested as violaceous, scaly and atrophic plaques affecting hands, cold-induced ulcerative distal lesions and erythemato-violaceous nodules on the cheeks, ears and nose, nail dystrophy, distal digital gangrene, and nasal septum perforation. Other cutaneous lesions such as telangiectasia, pustules, blisters, erythematous plaques may also occur, mostly on acral sites (14).

##### Cutaneous histopathology

Dermatopathology shows medium and small-vessel vasculitis with dense neutrophilic infiltrates and karyorrhexis in the vessel wall, as well as fibrin endovascular microthrombi (32, 151). Biopsies of telangiectatic plaques show perivascular infiltration by lymphocytes and neutrophils with leukocytoclasia, without involvement of the vessel walls (14).

#### Familial Chilblain Lupus

Familial chilblain lupus or TREX1-associated systemic lupus erythematosus is an autosomal dominant autoinflammatory disease caused by either loss-of-function mutations in *TREX1* and *SAMHD1* genes or gain-of-function mutations in the *TMEM173* gene, both leading to type I IFN overproduction (152–154).

Clinical manifestations consist of early-onset of mucocutaneous lesions and arthralgia, with occasional periodic fever and infrequent increased inflammatory markers. Low-titer autoantibodies, including ANA and anti-C1q-autoantibodies are usually present. Successful treatment with JAK inhibitors has been described (153, 155).

##### Dermatologic manifestations

Patients present with cold-induced chilblain lesions at acral locations (fingers, toes, nose, and ears) with subsequent ischemia and ulceration of these regions. Nails can show dystrophy or onychomadesis. Nailfold capillaroscopy may appear with irregular capillary loops and tortuous appearance. Livedo reticularis, malar rash, photosensitivity, and oral and nasal ulcers have also been described (152–154).

##### Cutaneous histopathology

Histological examination of skin reveals perivascular lymphohistiocytic infiltrates along with expression of the type I IFN-induced myxovirus resistance protein A within the endothelial cells (153).

#### Aicardi-Goutières Syndrome (AGS)

AGS comprise a group of seven monogenic autoinflammatory diseases, most of which are inherited with an autosomal recessive pattern, caused by mutations in several genes encoding proteins involved in intracellular degradation or sensing of nucleic acids. *TREX1, RNASEH2A, RNASEH2B, RNASEH2C, SAMHD1, ADAR1, IFIH1*, and *DDX58* are the genes involved in AGS. Mutations in these genes induce high levels of IFNα, both in blood and cerebrospinal fluid, which are thought to be responsible for systemic and cerebral tissue damage (156). In addition, dyschromatosis symmetrica hereditaria is an autosomal dominant skin disease caused by mutations in the *ADAR1* gene consisting of hyper- and hypo-pigmented macules on the dorsal aspects of the extremities. Patients with homozygous or compound heterozygous *ADAR1* mutations may present with a combination of AGS6 and dyschromatosis symmetrica hereditaria (157).

All forms of AGS share several features in common, such as a neonatal-onset encephalopathy consisting in basal ganglia calcifications, spasticity, dystonia, progressive cerebral atrophy, and microcephaly, as well as fever and hepatosplenomegaly (14). Abnormal laboratory results include lymphocytosis and elevated IFNα levels in cerebrospinal fluid. Patients may develop some autoimmunity features resembling systemic lupus erythematosus, such as arthritis, lymphopenia, thrombocytopenia, and ANA positivity (156).

No traditional treatment options, including glucocorticoids and conventional immunosuppressants and anti-cytokines agents, are useful. As for previous interferonopathies, JAK inhibitors seem also to control AGS activity (145, 156, 158, 159).

##### Dermatologic manifestations

Skin involvement comprises chilblain lesions on the feet, hands, and ears, digital vasculitis, generalized skin mottling, lipoatrophy, panniculitis, and acral necrotic lesions (14, 156, 160).

##### Cutaneous histopathology

No data regarding cutaneous histopathology is available in AGS.

#### Spondyloenchondrodysplasia With Immune Dysregulation (SPENCDI)

SPENCDI is an autosomal recessive autoinflammatory disease caused mutations in the *ACP5* gene encoding tartrate-resistant phosphatase. The lack of activity of this enzyme leads to a constitutive gain-of-function of osteopontin, a multifunctional protein involved in bone remodeling and immune regulation causing autoimmunity through a type I interferon expression signature (161).

SPENCDI is clinically characterized by bone dysplasia with subsequent growth retardation, and neurologic manifestations, such as cerebral atrophy, intracranial calcifications, seizures, and spastic paraparesis. Systemic and organ-specific autoimmune diseases are commonly present. These include systemic lupus erythematosus, antiphospholipid syndrome, Sjögren syndrome, Raynaud's disease, inflammatory myositis, arthritis, vitiligo, hypothyroidism, hemolytic anemia, and thrombocytopenia. Consequently, autoimmune markers are also frequently present, including positive ANA, anti-DNA antibodies, and hypocomplementemia (161).

Glucocorticoids, chloroquine, and other additional immunosuppressive agents, such as cyclophosphamide, azathioprine, mycophenolate mofetil, and rituximab have been used with good results (161).

##### Dermatologic manifestations

Cutaneous manifestations include severe eczema, Raynaud's phenomenon, distal sclerodermatous/acrocyanotic changes, and leukocytoclastic vasculitis presenting with purpuric lesions. Livedo reticularis and occlusive vasculitis leading to digital auto-amputation have also been described. Capillaroscopy may reveal edema and sludging or disappearance of parallel loops of some dilated capillaries (161).

##### Cutaneous histopathology

The skin biopsy from a patient with SPENCDI confirmed a non-specific leukocytoclastic vasculitis with perivascular neutrophilic infiltrate, without deposition of complement or immunoglobulin at direct immunofluorescence (161).

### Hyperkeratotic Lesions

#### NLRP-1 Associated Disease (NAIAD)

NAIAD is an autoinflammatory disease inherited with a recessive or dominant pattern due to mutations in the *NLRP1* gene, which encodes NLRP1 protein. NLRP1 is the central inflammasome in the skin. Mutations in PYRIN or LRR domains lead to constitutive NLRP1 inflammasome activation and IL-18 production (138). NAIAD is currently categorized as AIKD (103).

Patients present with infantile-onset attacks of recurrent fever lasting 3–4 days, accompanied by hyperkeratotic lesions, polyarticular arthritis and chronic relapsing infections. Blood tests show high CRP levels during flares, low-titer of ANA, vitamin A deficiency, and raised transitional B cells (162). Treatment with vitamin A and acitretin has been associated with clinical improvement.

##### Dermatologic manifestations

Most patients show disseminated erythematous follicular hyperkeratosis. Cases of familial keratosis lichenoides chronica (also considered an AIKD), associated with multiple self-healing palmoplantar carcinoma, as well as larynx involvement resembling human papillomavirus infection have been reported in NAIAD patients (162, 163).

##### Cutaneous histopathology

Skin biopsy shows orthokeratotic hyperkeratosis with papillomatosis, acanthosis and hypergranulosis. Numerous dyskeratotic cells sparse throughout the epidermis, without involving the basal layer, have been observed (162).

### Hyperpigmented Lesions

#### H Syndrome

H syndrome is an autosomal recessive autoinflammatory disease caused by mutations in the *SLC29A3* gene encoding ENT3 (164).

This syndrome is referred to as “H syndrome” to describe some of the disease hallmarks: hyperpigmentation, hypertrichosis, hepatosplenomegaly, heart anomalies, hearing loss, and hypogonadism. Therefore, the disease is clinically characterized by progressive sclerotic skin lesions, cardiac anomalies, short stature, and childhood-onset sensorineural hearing loss. Other manifestations include scrotal masses, azoospermia, hepatosplenomegaly, micropenis, dilated scleral vessels, exophthalmos, facial telangiectasia, and camptodactyly (164, 165). Laboratory tests reveal high ESR values and growth hormone deficiency (164).

Most treatments, including glucocorticoids, colchicine, cytotoxic immunosuppressants, IFNα, anakinra, canakinumab, adalimumab, and radiotherapy, are associated with an inadequate response (164).

##### Dermatologic manifestations

Cutaneous involvement starts with progressive sclerotic, hyperpigmented plaques on the lower limbs with hypertrichosis, and less frequently with ichthyotic desquamation. These lesions usually appear on the inner aspect of the thighs and progress to the abdomen, genitalia, ankles and feet, with sparing of the knees and buttocks. The presence of plaques in axillae and trunk is infrequent (164, 165).

##### Cutaneous histopathology

Dermatopathology is characterized by an increase of melanocytes with acanthosis in the basal layer and sclerodermiform changes with interstitial macrophagic infiltrates in the dermis and fat tissue. Perivascular infiltrates of lymphocytes, mast cells and plasma cells are also noted. Emperipolesis (cell engulfment phenomena) may be occasionally observed (22, 165).

### Bullous Lesions

#### Autoinflammation and PLCγ2-Associated Antibody Deficiency and Immune Dysregulation (APLAID)

APLAID is an autosomal dominant autoinflammatory disease caused by two missense mutations (S707Y and L848P) in the *PLC*γ*2* gene. Despite being produced by the same gene that PLAID, mutants in APLAID result in hyperactivation of PLCγ2 signaling pathway and are associated with a different disease phenotype (71, 166).

APLAID patients present with early-onset recurrent attacks of blistering skin lesions, eye inflammation with ocular hypertension, inflammatory bowel disease, arthralgia, and sinopulmonary infections caused by a mild humoral immunodeficiency. Acute phase reactants tend to be normal and switched memory B-cells are almost absent. Contrary to PLAID, APLAID is not characterized by cold-induced urticaria and the presence of circulating autoantibodies (71).

While TNF-blockers have not shown efficacy, high-dose glucocorticoids, and anakinra have been reported to control inflammatory symptoms (71).

##### Dermatologic manifestations

Early-onset recurrent blistering lesions resembling epidermolysis-bullosa is the most common picture. Later in life, these lesions tend to evolve to recurrent erythematous plaques and vesiculopustular lesions that get worse with sun and heat exposure. Cellulitis-like plaques and granulomata, as well as cutis laxa, have also been described (70, 71, 166). More recently, three cases with different presentation have been reported: a newborn patient with generalized erythematous papules evolving to vesicles, pustules, and crusts involving face, gluteal region, and extremities (167) and two patients of 6 and 14 years-old with a papulovesicular skin rash with granuloma formation and cutis laxa (168).

##### Cutaneous histopathology

A biopsy from a plaque lesion showed a dense dermal interstitial and perivascular mixed inflammatory infiltrate composed of lymphocytes, histiocytes, eosinophils, and karyorrhectic nuclear debris (71).

### Aphthous Lesions

#### Haploinsufficiency of A20 (HA20)

HA20, also known as monogenic Behçet-like disease, is an autosomal dominant autoinflammatory disease caused by mutations in the *TNFAIP3* gene encoding protein A20. Mutated A20 results in increased NF-κB signaling and NLRP3 hyperactivity (11, 169).

The clinical picture of HA20 is characterized by the triad of orogenital ulcers, ocular inflammation and non-deforming polyarthritis (170). Abdominal pain, pharyngitis, pericarditis, retinal vasculitis, and central nervous system vasculitis are also frequent manifestations (32, 171). Several organ-specific and systemic autoimmune diseases have been associated with HA20. Some of them include Hashimoto thyroiditis, type 1 diabetes mellitus, neutrophilic dermatosis, erythema nodosum, pseudofolliculitis, central nervous system vasculitis, Kawasaki disease, IgA vasculitis, nephrotic syndrome, idiopathic thrombocytopenic purpura, or interstitial lung disease. Acute phase reactants are increased during flares and low-titer autoantibodies may be present in cases with autoimmune diseases (171).

Treatment with colchicine, glucocorticoids, methotrexate and thalidomide may be useful. Refractory cases to previous drugs have been reported to respond to anti-TNFα, anti-IL-1, and anti-IL-6 agents (170, 171).

##### Dermatologic manifestations

**Figure 11:**
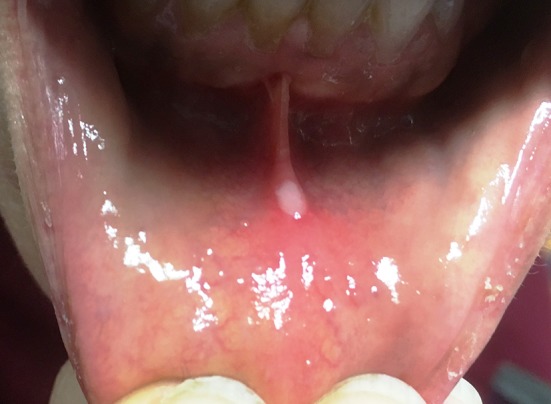
Oral aphtous lesion in a patient with HA20. Written informed consent was obtained from the patient for the publication of this image.

Oral, genital, and gastrointestinal non-scarring ulcers are the most frequent manifestations (Figure 11). Skin involvement is characterized by pustular or vesicular rashes, acne, dermal abscesses, mild desquamation, and hyperkeratosis. Pathergy test can be positive in some patients (170, 171).

##### Cutaneous histopathology

Histological data of the skin is limited. The presence of an epidermal infiltrate of lymphocytes and neutrophils with extensive intradermal mucin accumulation and scarce inflammatory infiltrates has been reported (171).

#### Autoinflammatory Periodic Fever, Immunodeficiency, and Thrombocytopenia (PFIT)

PFIT is an autoinflammatory disease caused by a homozygous missense mutation in the actin regulatory gene *WDR1*, which encodes WDR1. Mutant WDR1 is thought to facilitate assembly of pyrin inflammasome, leading to excessive IL-18 production (172).

PFIT clinical features include recurrent fever attacks, lasting from 3 to 7 days and with 6–12 weeks periodicity. Fever is accompanied by oral ulcers, intermittent thrombocytopenia and cellular immunodeficiency, increasing the rate of infections. Raised acute phase reactants, leukocytosis, hyperferritinemia, and thrombocytopenia are observed during attacks (172).

Glucocorticoids, colchicine, conventional immunosuppressive drugs, and anakinra have been associated with poor responses. A case treated with an allogeneic hematopoietic stem cell transplantation has been reported with success (172).

##### Dermatologic manifestations

The most critical skin manifestation is the presence of severe oral ulcers and inflammation that cause scarring and microstomia (172).

##### Cutaneous histopathology

There are no reports regarding histopathological features in PFIT skin lesions.

#### C/EBPε-Associated Autoinflammation and Immune Impairment of Neutrophils (CAIN)

CAIN is an autosomal dominant autoinflammatory disease caused by mutations in the *CEBPE* gene encoding the transcription factor C/EBPε, which regulates both the inflammasome and the interferome.

CAIN is characterized by a combination of autoinflammation, immunodeficiency and neutrophil dysfunction. Disease onset has been reported during adolescence and tends to subside after menopause. The clinical presentation consists of periodic attacks of abdominal pain and high fever during 4–5 days, every 2–4 weeks. Other manifestations during attacks include oral ulcers, cutaneous abscesses, pyoderma gangrenosum, intra-abdominal granulomas, and upper respiratory tract infections. Mild bleeding diathesis with frequent nosebleeds and hematomas after needle sticks and surgical procedures have also been described. ESR elevation is frequent.

In CAIN patients, blockade of IL-1β and anti–IL-18 are candidate therapies, still untested (173).

##### Dermatologic manifestations

Crater-like buccal ulcers are the most frequent mucocutaneous features. Severe recurrent tongue, gluteal, submandibular abscesses, purulent paronychia, pyoderma gangrenosum, and wounds with delayed healing have also been described.

##### Cutaneous histopathology

No information about dermatopathologic features in CAIN lesions has been reported.

#### NFKB1-Associated Autoinflammatory Diseases (NFKB1-AD)

NFKB1-AD comprise a group of three different autosomal dominant diseases due to mutations in the *NFKB1* gene. These mutations affect the NK-κB subunits p50 and p105, resulting in an increased expression of IL-1β and TNF in some cases (174).

Initial descriptions of patients with *NFKB1* gene mutations were associated with an immunodeficiency phenotype consisting of recurrent respiratory tract infections leading to chronic lung disease with bronchiectasis, diarrhea, lymphadenopathy, splenomegaly, recurrent autoimmune phenomena (hemolytic anemia, thrombocytopenia, and leukopenia), hypogammaglobulinemia, deficient production of specific antibodies, and decreased class-switched and memory B cells (175, 176).

Subsequently, two additional autoinflammatory phenotypes associated to different mutations in the *NFKB1* gene have been described in two families (177). The first autoinflammatory phenotype is NFKB1-associated Behçet-like disease, which has been associated with the non-truncating mutation H67R in the *NFKB1* gene. It was described in six individuals within the same family presenting with clinical manifestations similar to those observed in Behçet disease (mucosal ulcers, arthritis, and abdominal pain) (177). Notably, mutations in *NFKB1* affect the same pathway as in HA20. However, Behçet-like disease associated with NFKB1 mutations was also associated with IgG-hypogammaglobulinemia, depletion of switched memory B cells and increased susceptibility to respiratory tract infections, thus overlapping somewhat with the immunodeficiency and autoimmunity phenotype described first for NFKB1-associated disease (175, 176). Behçet-like phenotype seems not to cause canonical inflammasome overactivation *in vitro*, thus targeting IL-1β and TNF might not be useful (177).

The second autoinflammatory phenotype is NFKB1-associated sterile familial autoinflammatory necrotizing fasciitis (FANF), which is caused by the truncating mutation R157X in the *NFKB1* gene. It was described in two brothers who presented with recurrent, sterile, isolated necrotizing inflammation after tissue trauma caused by minor surgery, and rapidly extending into muscle fasciae, thus corresponding to necrotizing fasciitis. Patients had no other organ or systemic involvement nor any obvious manifestations of immunodeficiency (177). This mutation caused increased inflammasome activation *in vitro*, suggesting that agents targeting IL-1β or TNF might be useful in such autoinflammatory necrotizing fasciitis patients (174).

##### Dermatologic manifestations

The most common cutaneous lesions in patients with NFKB1-associated Behçet-like disease are mucosal aphthae affecting oral mucosa, esophagus, and genitalia. Lesions consisting of postoperative deep necrotizing fasciitis have been described in two patients of the same family with FANF (177). Of note, NFKB1-associated FANF lesions have been included in the section of pustular, pyogenic or neutrophilic dermatosis-like rashes in Table 2.

##### Cutaneous histopathology

Information about genital aphthae biopsy displayed a small vessel vasculitis, similar to that seen in Behçet's patients (177).

#### RELA Haploinsufficiency

RELA haploinsufficiency is an autosomal dominant autoinflammatory disease caused by mutations in the *RELA* gene, which encodes RelA, a subunit of NF-κB. The heterodimer RelA/NF-κB1 constitutes the predominant form of NF-κB, critical for cell survival. A biallelic requirement for RelA in order to maintain the normal cell function in stromal and epithelial cells, which is essential for mucosal integrity, has been reported. However, lymphocyte function is preserved in mice with RELA haploinsufficiency. This would explain why these patients with an impaired NF-κB signaling and an increased sensitivity to TNF have mucosal abnormalities without immunodeficiency (178).

Clinically these patients present with mucosal ulcers and gastrointestinal symptoms, such as abdominal pain, vomiting, and diarrhea, which can resemble an inflammatory bowel disease. Fever and elevated acute phase reactants are also present.

Treatment with glucocorticoids and TNF-α inhibitors have shown efficacy (178).

##### Dermatologic manifestations

Oral and/or genital ulcers are the most common mucocutaneous feature described in patients with RELA haploinsufficiency (178).

##### Cutaneous histopathology

No information regarding cutaneous histopathological features in RELA haploinsufficiency has been reported yet.

#### Monogenic Forms of Inflammatory Bowel Disease

Crohn's disease and ulcerative colitis are considered inflammatory bowel diseases (IBD), diseases with a polygenic or multifactorial etiology, in which complex interactions between genetic and environmental factors play an important role. Although over 150 genetic loci are associated with IBD, the genetic contribution toward heritability of the majority of those loci is low. However, recent studies have reported an increasing spectrum of human monogenic diseases that can present with IBD-like intestinal inflammation. Most of patients with those genetic defects present with very early onset IBD (during early childhood). However, as occur in polygenic IBD, in the monogenic forms, a variable penetrance of the clinical phenotype has been similarly described, also suggesting a role for modifier genes and/or gene–environmental interactions (179).

Oral aphthae may occur in polygenic and monogenic forms of IBD. With regard to monogenic forms, IL-10 signaling defects associated with very early onset IBD is an autosomal recessive monogenic autoinflammatory disease caused by mutations in genes encoding IL-10 and IL-10-receptor. Clinical manifestations start within the first 3 months of life and include bloody diarrhea, abscesses, perianal fistula, folliculitis, oral aphthous lesions and arthritis. The intermittent course of colitis with deep ulcerations is also indistinguishable from that of Crohn disease (179).

## Conclusions

Cutaneous inflammatory lesions are commonly present in most of monogenic autoinflammatory diseases. Among them, non-specific maculopapular rashes and urticarial lesions are the most frequent manifestations, which have some differential traits regarding similar lesions without an autoinflammatory cause. While the evidence of systemic involvement will draw the attention toward an autoinflammatory origin, a genetic test showing pathogenic mutations in causal genes will confirm the diagnosis of a monogenic autoinflammatory disease.

Because the information regarding skin manifestations is still scarce, this review analyzes the most relevant histopathological and clinical features of cutaneous involvement in monogenic autoinflammatory diseases and groups the diseases based on the predominant cutaneous lesions, which were divided in: (1) maculopapular rashes or inflammatory plaques; (2) urticarial rashes; (3) pustular, pyogenic, or neutrophilic dermatosis-like rashes; (4) panniculitis or subcutaneous nodules; (5) vasculitis or vasculopathy; (6) hyperkeratotic lesions; (7) hyperpigmented lesions; (8) bullous lesions; and (9) aphthous lesions.

Therefore, the classification based on the predominant skin lesion in patients in whom a monogenic autoinflammatory disease is suspected may be a supporting tool to guide final diagnosis.

## Author Contributions

IF-N wrote and reviewed the article and provided most of the images for the article. JM collaborated with the writing, reviewed the manuscript, and provided some images. XS reviewed the article and contributed with useful corrections and some images. JH-R assisted with the writing, extensively reviewed the manuscript, and provided some images.

### Conflict of Interest

The authors declare that the research was conducted in the absence of any commercial or financial relationships that could be construed as a potential conflict of interest.

## Abbreviations

AIKD: Autoinflammatory keratinization diseases
ANA: Antinuclear antibodies
ANCA: Antineutrophil cytoplasmic antibodies
AGS: Aicardi-Goutières syndrome
APLAID: Autoinflammation and PLCγ2-associated antibody deficiency and immune dysregulation
CAIN: C/EBPε-associated autoinflammation and immune impairment of neutrophils
CANDLE: Chronic atypical neutrophilic dermatitis with lipodystrophy and elevated temperature syndrome
CAPS: Cryopyrin-associated periodic syndrome
CINCA: Chronic infantile, neurologic cutaneous and articular
CPR: C-reactive protein
CRMO: Chronic recurrent multifocal sterile osteomyelitis
DADA2: Deficiency of adenosine deaminase 2
DIRA: Deficiency of IL-1 receptor antagonist
DITRA: Deficiency of IL-36 receptor antagonist
EMA: European Medicines Agency
ESR: Erythrocyte sedimentation rate
FANF: Familial autoinflammatory necrotizing fasciitis
FCAS: Familial cold autoinflammatory syndrome
FDA: Food and Drug Administration
FMF: Familial Mediterranean fever
HA20: Haploinsufficiency of A20
HIDS: Hyper-IgD syndrome
Ig: Immunoglobulin
IL: Interleukin
IBD: Inflammatory bowel disease
IFN: Interferon
LUBAC: Linear ubiquitination chain assembly complex
MA: Mevalonic aciduria
MAS: Macrophage activation syndrome
MAVS: Mitochondrial antiviral signal
MKD: Mevalonate kinase deficiency
MVK: Mevalonate kinase
MWS: Muckle-Wells syndrome
NAIAD: NLRP-1 associated disease
NLRC4-AD: NLRC4-associated autoinflammatory diseases
NOMID: Neonatal-onset multisystem inflammatory disease
NSAID: Non-steroidal anti-inflammatory drugs
ORAS: OTULIN-related autoinflammatory syndrome
PAAND: Pyrin-associated autoinflammation with neutrophilic dermatosis
PAPA: Pyogenic sterile arthritis pyoderma gangrenosum and acne
PFAPA: Periodic fever, aphthous stomatitis pharyngitis and cervical adenitis
PFIT: Autoinflammatory periodic fever, immunodeficiency and thrombocytopenia
PLAID: PLCγ2-associated antibody deficiency and immune dysregulation
SAA: Serum amyloid protein
SAPHO: Synovitis, acne, pustulosis hyperostosis and osteitis
SAVI: STING-associated vasculopathy with onset in infancy
STING: Stimulator of IFN genes
SMS: Singleton–Merten syndrome
sJIA: Systemic juvenile idiopathic arthritis
SPENCDI: Spodyloenchondrodysplasia with immune dysregulation
TNF: Tumor necrosis factor
TACE: TNF-α converting enzyme
TRAPS: TNF receptor-associated periodic syndrome
TLR: toll-like receptors.
